# Airway management for patients with ossification of the anterior longitudinal ligament of the cervical spine

**DOI:** 10.1186/s40981-015-0002-9

**Published:** 2015-12-31

**Authors:** Miki Iida, Kumiko Tanabe, Shuji Dohi, Hiroki Iida

**Affiliations:** Department of Anesthesiology and Pain Medicine, Gifu University Graduate School of Medicine, Yanagido 1-1, Gifu, 501-1194 Japan

**Keywords:** Ossification of the anterior longitudinal ligament, Forestier’s disease, Diffuse idiopathic skeletal hyperostosis, Difficult intubation

## Abstract

Ossification of the anterior longitudinal ligament (OALL), also called Forestier’s disease or diffuse idiopathic skeletal hyperostosis, is characterized by anterior bridging osteophytes of unknown etiology. OALL may cause dysphagia, dyspnea, dysphonia, and acute airway obstruction. We report difficulty in tracheal intubation during anesthesia induction in two OALL patients. In an 82-year-old man, anterior bridging osteophytes (of the cervical region) were observed on preoperative lateral radiograph after several attempts of tracheal intubation for the operation of the anterior fusion of cervical spine. During the same procedure in another 69-year-old man, fiberoptic-assisted awake intubation was extremely difficult because of posterior hypopharyngeal wall protuberance by osteophytes of cervical spine; although tracheal intubation for anesthesia was uneventful on two previous occasions over the months. OALL is usually asymptomatic, but it has been found in 12 % of autopsies and may exaggerate with age. Dysphagia, difficulties with tracheal and/or gastric intubation, acute respiratory compromise, and sleep apnea result from the presence of cervical osteophytes. Anesthesiologists should be aware that tracheal intubation for such patients may be difficult, and thus the preoperative evaluation and airway management need careful consideration.

## Background

Ossification of the anterior longitudinal ligament (OALL), also called Forestier’s disease or diffuse idiopathic skeletal hyperostosis (DISH), is a non-inflammatory disease characterized by the presence of anterior bridging osteophytes of unknown etiology. Although it is asymptomatic in some cases, this disease may produce dysphagia, dyspnea, dysphonia, and (exceptionally) acute airway obstruction [1]. Difficulties in tracheal intubation, as well as aspiration pneumonia and spinal cord injury, are potential problems in anesthetic management. Herein, we report two patients with OALL in which airway problems occurred during the induction of anesthesia and tracheal intubation.

## Case presentation

**Fig. 1:**
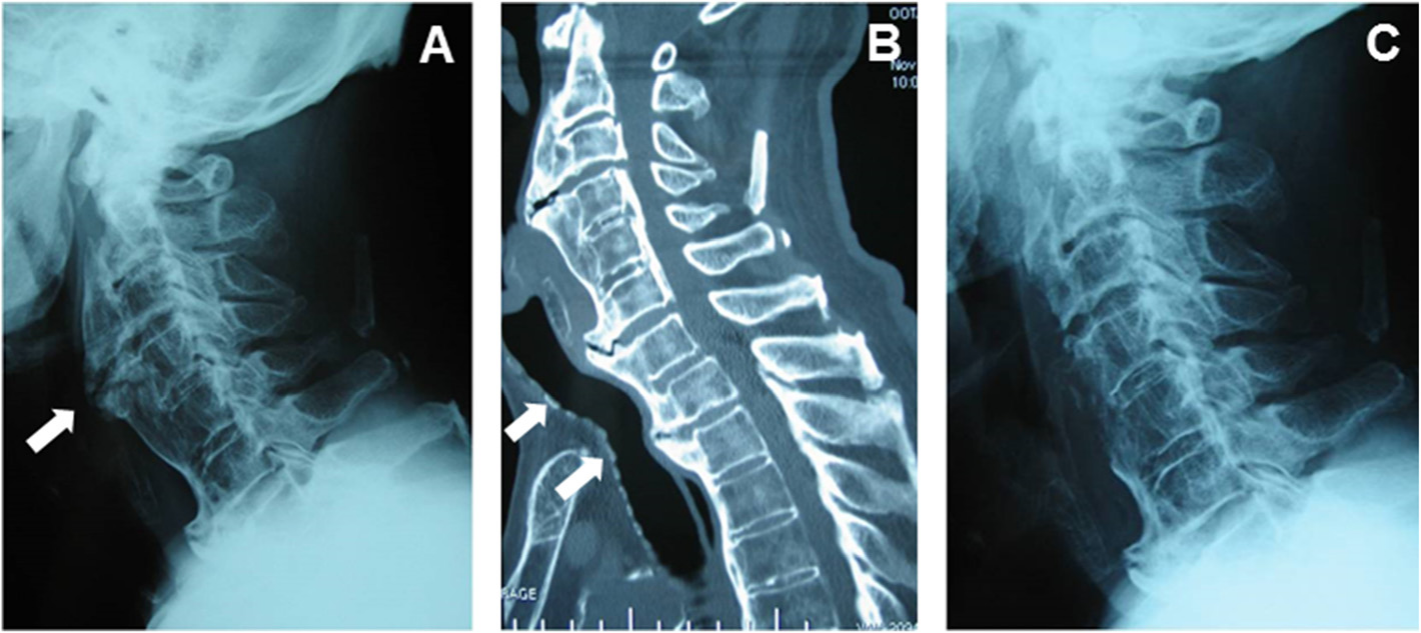
**a** Preoperative lateral cervical spine radiograph showing extensive ossification of anterior longitudinal ligament (*white arrow*) in an 82-year-old man. **b** Preoperative sagittal CT showing hyperostosis, mostly C2–C4 and C6–C7, producing anterior laryngopharyngeal displacement, with angulation of the trachea distally (*white arrows*), in an 82-year-old man. **c** Postoperative lateral cervical spine radiograph showing that the trachea was no longer compressed and had straightened in an 82-year-old man

An 82-year-old man with OALL was scheduled for resection of the osteophytes by left anterolateral cervicotomy. He had a history of hypertension and had been experiencing repeated aspiration pneumonia for several years. There was no history of respiratory distress or obstructive sleep apnea, but his family had noticed him snoring. Upon physical examination, his neck movements were not grossly restricted and his mouth opening was determined to be within the normal range. Since he had lost all his teeth, the pharyngeal view was Mallampati Class I. The chest radiogram was normal apart from an old inflammatory change in the right lower lung. On arrival in the operating room, he was hemodynamically stable without anesthetic premedication, and his oxygen saturation was 96 % on room air. After his lungs had been preoxygenated with 100 % O_2_, general anesthesia was induced with intravenous thiopental and fentanyl. After muscle relaxation had been induced using vecuronium, direct laryngoscopy revealed that his Cormack Lehane score was Class I. An 8.5-mm endotracheal tube (internal diameter [I.D.], 8.5 mm; outside diameter [O.D.], 11.5 mm; Mallinckrodt, Athlone, Ireland) could not be advanced through the subglottic region, pooling of saliva was found within the piriform sinus. After two trials, with the same result, the tube size was changed to 7.5 mm I.D., and this could be advanced with slight resistance. However, it was difficult to insert the nasogastric tube. Because a subglottic tracheal stenosis was suspected, his clinical records were reevaluated. According to his medical chart, he had taken only liquid food for several months because of dysphagia. Moreover, a lateral cervical radiogram and cervical computerized tomography (CT) scan revealed hyperostosis, mostly of C2–C4 and C6–C7, producing anterior laryngopharyngeal displacement, with angulation of the trachea distally (Fig. 1a, b). It seemed likely that his angulation caused the difficulty experienced during intubation. At the end of the 80-min surgery, which was uneventful, we recognized that his trachea was no longer compressed and had straightened (Fig. 1c). Subsequently, his trachea was extubated with no problems, and he was taken wide awake to the intensive care unit (ICU).

A 69-year-old man was scheduled for an off-pump coronary artery bypass graft (CABG) by way of a left anterolateral thoracotomy. His chief complaint was dysesthesia of the left arm caused by ossification of the posterior longitudinal ligament (OPLL). However, his preoperative ECG suggested an old antero–septal myocardial infarction, and subsequent coronary angiography revealed occlusion of both the left anterior descending branch and left circumflex artery, so CABG was planned first. He had a history of hypertension and renal insufficiency (creatinine clearance 49.9 mL/min). Upon physical examination, his neck movement was slightly restricted, but his dysesthesia was not made worse by neck movement. He could open his mouth widely, without limitation, and his Mallampati score was Class II. The chest radiograph was unremarkable. In the operating room, after the establishment of noninvasive monitors (five-lead ECG and pulse oximeter), an arterial catheter was inserted at the left radial artery for arterial blood pressure monitoring, and general anesthesia was induced with intravenous midazolam and fentanyl. After muscle relaxation had been induced using vecuronium, direct laryngoscopy was performed, and we assigned a Cormack Lehane score of Class II. We succeeded with left bronchial intubation at the first attempt. Insertion of transesophageal echocardiography was easy, too. The procedure was completed uneventfully. His vital signs were stable, and he was transported to the ICU under sedation. His postoperative course was uneventful.

Two months after the CABG, he was scheduled for posterior fusion of the cervical spine. After general anesthesia had been induced using thiopental and fentanyl, and muscle relaxation using vecuronium, direct laryngoscopy was performed. However, only the epiglottis could be seen. We therefore decided to use an airway scope (AWS; Pentax, Tokyo, Japan), and by this means, visualization of his vocal cords was achieved easily. After the surgical procedure had been completed, his endotracheal tube was extubated in the operation room without airway problems.

**Fig. 2:**
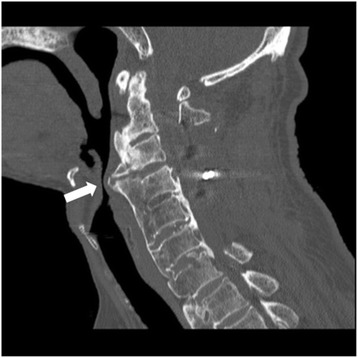
Sagittal CT showing overhang of posterior hypopharyngeal wall by osteophytes (*white arrow*) in a 69-year-old man

One month later, he was scheduled for resection and grinding of osteophytes via an anterior cervicotomy. As a result of the posterior fusion, his neck movement was grossly restricted, compared to that seen at the time of the previous operation. Therefore, fiberoptic-assisted awake intubation was planned. Following a gargle with 4 % lidocaine, the pharynx was anesthetized using a tracheal spray tube. Percutaneous transtracheal anesthesia with 2 % lidocaine was also employed. After preoxygenation of 100 % O_2_, nasotracheal fiberoptic intubation was attempted, but only the epiglottis could be visualized. The bump of the posterior hypopharyngeal wall was displaced anteriorly and was bulging into the hypopharyngeal lumen. Since the epiglottis was veiling his vocal cords, the fiberscope could not be advanced toward the trachea, and it took 1 h to accomplish tracheal intubation. An anatomical abnormality was suspected, and so his preoperative image was reviewed. CT with multiplanar reformatted imaging revealed osteophytes overhanging the posterior hypopharyngeal wall (Fig. 2). At the end of surgery, a lateral cervical radiogram revealed that the osteophytes had been removed. He was mechanically ventilated in the ICU under sedation, and extubated uneventfully the next morning.

## Discussion

Ossification most notably affects the anterior longitudinal ligament of the spine; a condition called OALL. It mostly affects men, and the mean age of the patients is 60 years [2]. The thoracic region is most commonly affected, followed by the lumbar and cervical regions [2]. Ossification does not always occur solely in the vertebral column, it can occur in other parts of the musculoskeletal system too [2]. There are three radiologic criteria for the diagnosis of OALL: osseous bridging of at least four contiguous vertebral bodies, preservation of intervertebral disc height, and absence of sacroiliac or apophyseal joint ankylosis [3]. A number of possible causal factors of metabolic, environmental, or endocrine origin have been investigated, but no conclusive results have been obtained [2, 3].

OALL has been reportedly found in 12 % of autopsies, but it is generally asymptomatic [2, 3]. Indeed, it is often discovered coincidentally as a remarkable radiologic finding when a radiograph is solicited for the other disease [2]. Dysphagia, difficulties with tracheal and/or gastric intubation, acute respiratory compromise, and sleep apnea has been reported to result from the presence of cervical osteophytes [2–4]. The cervical hyperostosis associated with OALL may lead to symptoms resulting from compression and/or distortion of the airway and/or the alimentary tract [4]. Complications from these cervical osteophytes are rare, although they can occasionally be severe, even life-threatening [1]. The level of the cervical spine at which osteophytes form has been postulated to correlate with the presenting symptoms. The most commonly involved vertebrae are C4–C5, followed by C5–C6 and C3–C4 [1]. Lower cervical vertebrae, around the levels of C4, C5, and C6, may impinge on the esophagus and distal trachea, where it is tethered at the level of the cricoids, whereas the upper cervical spine may impinge more on the oropharynx, causing globus, stridor, or respiratory compromise [1]. Although cervical radiographs may show cervical osteophytes, enhanced CT with multiplanar reformatted has proved to be the best diagnostic method for the localization of laryngeal compression and can be of help in the planning of anesthesia [4]. If laryngeal compression is suspected, a preoperative sagittal CT is also helpful for the evaluation of airway obstruction and for the planning of tracheal intubation. Our two cases suggest that although the difficulties in tracheal intubation resulting from OALL are rare, anesthesiologist should suspect the presence of OALL, whenever they encounter unexplained difficulty in airway management in a patient who has no airway signs and/or no remarkable previous anesthesia history.

For patients with OALL, fiberoptic-assisted awake intubation is perhaps the safest choice when difficult intubation is suspected [5, 6]. Since great difficulty in tracheal intubation may occur secondary to anterior bulging of the posterior pharyngeal wall, anterior laryngeal displacement, angulation of the trachea distally and neck stiffness [3], and hyperostosis on the ligaments around the larynx [7], careful evaluation of the epiglottis during laryngoscopic examination is mandatory if such difficulties are to be avoided. In fact, tracheotomy may be indicated in such cases to avoid a catastrophic event.

Furthermore, osteophytes are likely to be missed in routine airway examination by frontal radiography. Therefore, it is necessary that preoperative radiography is evaluated including the lateral view, if the patient has diagnosis of OALL. And airway management of OALL patients needs careful consideration and modifications made if necessary, dependent upon progress, in each case.

## Consent

Written informed consents were obtained from the patients for publication of this case report and any accompanying images. A copy of the written consent is available for review by the Editor-in-Chief of this journal.
